# Diagnosis and Surgical Management of a Hemorrhagic Hepatic Pseudocyst in a Young British Shorthair Cat

**DOI:** 10.3390/vetsci13101000

**Published:** 2026-09-23

**Authors:** Yerin Lee, Jong-O Kim, Min-Kwan Han, Hwi-Yool Kim, Jung-Moon Kim

**Affiliations:** 1 Department of Veterinary Surgery, College of Veterinary Medicine, Konkuk University, Seoul 05029, Republic of Korea; 2 24h Thenaeun Animal Medical Center, Geoje-si 53256, Republic of Korea

**Keywords:** cat, hepatic cyst, hepatic pseudocyst, intracystic hemorrhage, surgical excision, histopathology

## Abstract

Hemorrhagic hepatic pseudocysts are uncommon in cats and can present diagnostic and therapeutic challenges. This report describes the diagnosis and surgical management of a massive hemorrhagic hepatic pseudocyst in a 1-year-old intact male British Shorthair cat presented for castration with several days of intermittent vomiting. Physical examination revealed marked abdominal distension, while routine hematologic and biochemical testing showed no clinically significant abnormalities. Abdominal radiography revealed a large soft tissue opacity occupying most of the abdominal cavity. Ultrasonography identified a large fluid-filled cystic structure, and computed tomography confirmed a large hepatic cystic lesion with displacement of adjacent organs. Surgical exploration revealed a solitary hepatic cyst containing dark red hemorrhagic fluid. The cyst-fluid total bilirubin concentration was markedly higher than the serum concentration, although no gross communication with the biliary tract was identified during surgery. The lesion was completely excised while preserving the adjacent hepatic parenchyma. Histopathologic examination revealed a fibrous cyst wall with extensive hemorrhage and multifocal necrosis without an epithelial lining, supporting the diagnosis of a hemorrhagic hepatic pseudocyst. At 26 months after surgery, the owner reported that the cat had remained clinically well, and abdominal radiographs showed no evidence of a recurrent abdominal mass. This case highlights hemorrhagic hepatic pseudocyst as a differential diagnosis for large hepatic cystic lesions in cats and suggests that complete surgical excision may be a treatment option for a resectable hepatic pseudocyst.

## Simple Summary

Hepatic cystic lesions are uncommon in cats, and hemorrhagic hepatic pseudocysts are particularly rarely reported. This report describes the case of a one-year-old British Shorthair cat that was presented with marked abdominal enlargement and intermittent vomiting. Diagnostic imaging revealed a very large fluid-filled lesion associated with the liver that occupied most of the abdominal cavity. The lesion was surgically removed and contained dark red hemorrhagic fluid. Microscopic examination of the removed tissue showed a fibrous wall with extensive bleeding and tissue damage but no epithelial lining, supporting a diagnosis of a hemorrhagic hepatic pseudocyst. The cat showed mild transient lethargy for several days after discharge but had no significant abnormalities at the 14-day postoperative recheck. At 26 months after surgery, the cat remained clinically well according to the owner, and follow-up abdominal radiographs showed no evidence of a recurrent abdominal mass. This case provides clinical information on the diagnosis, surgical management, and long-term outcome of a rare hemorrhagic hepatic pseudocyst in a cat.

## 1. Introduction

Hepatic cystic lesions are relatively uncommon in cats and can be classified as either congenital or acquired [1,2,3]. Congenital hepatic cysts are generally believed to arise from developmental abnormalities of the biliary system and may occur as multiple lesions, sometimes accompanied by renal cysts [1,3,4]. On the other hand, acquired hepatic cystic lesions are usually solitary [2]. Histopathologically, hepatic cysts are lined by biliary epithelium, whereas hepatic pseudocysts lack an epithelial lining [3,5]. Most hepatic cysts are asymptomatic until they become large enough to cause abdominal distension or compression of adjacent organs [1,2,6,7]. Reported clinical signs include abdominal distension, vomiting, dyspnea, and, less commonly, obstructive jaundice [1,6,7,8].

Hemorrhagic transformation is a rare complication of hepatic cysts and may complicate diagnostic interpretation because imaging findings can mimic cystic neoplasms [2,9,10,11]. Intracystic hemorrhage may alter the internal appearance of a cystic lesion on diagnostic imaging, further complicating characterization of the lesion [9,10,11]. Consequently, histopathologic examination remains essential for establishing a definitive diagnosis [2,9,10,11].

In cats, published reports have primarily described congenital hepatic cysts, polycystic liver disease, and vascular cysts [1,4,6,7,8,12], whereas hemorrhagic hepatic cystic lesions have been reported rarely in other felids [2]. Therefore, information regarding the clinical presentation, diagnostic findings, treatment, and outcome of hemorrhagic hepatic pseudocysts in domestic cats remains limited.

This report presents the diagnosis and surgical management of a massive hemorrhagic hepatic pseudocyst in a young British Shorthair cat.

## 2. Case Description

A 1-year-old intact male British Shorthair cat weighing 5.0 kg was presented for castration. The owner reported intermittent vomiting for several days before presentation. Marked abdominal enlargement was noted on physical examination, which had not been noticed by the owner.

Initial complete blood count and serum biochemical profiles were within reference intervals. No evidence of anemia was identified (hematocrit, 48.3%), and serum liver enzyme activities were within reference intervals (Table 1).

Radiographs revealed a large soft tissue opacity occupying most of the abdominal cavity, resulting in displacement of the gastrointestinal tract (Figure 1A,B). Abdominal ultrasonography identified a large hypoechoic fluid-filled cystic structure with a focal hyperechoic area within the lesion (Figure 1C). Computed tomography (CT) revealed a large cystic lesion arising near the liver, occupying a substantial portion of the abdominal cavity and displacing adjacent abdominal organs (Figure 2). The lesion showed homogeneous fluid attenuation, consistent with a hepatic cystic lesion.

The cat was hospitalized for a day before surgery for intravenous fluid therapy (0.9% sodium chloride, 2 mL/kg/h) and clinical monitoring. No additional clinical signs were observed during this period.

Surgical exploration was performed through a ventral midline celiotomy. A solitary cyst arising from the liver was identified intraoperatively (Figure 3A). The cyst contained dark red hemorrhagic fluid (Figure 3B), with no gross evidence of communication with the biliary tract. Analysis of the cyst fluid revealed a total bilirubin concentration of 3.38 mg/dL, markedly higher than the serum concentration (0.03 mg/dL; Table 1).

The cyst was completely excised using an electrothermal bipolar vessel-sealing device (LigaSure; Medtronic, Minneapolis, MN, USA) while preserving the adjacent hepatic parenchyma.

Postoperatively, meloxicam (0.05 mg/kg, SC) and gabapentin (3.5 mg/kg, PO, q12h) were administered for pain management, and amoxicillin/clavulanate (12.5 mg/kg, PO, q12h) was administered as antimicrobial therapy. The cat remained hospitalized for 2 days after surgery and received intravenous fluid therapy during hospitalization.

The excised lesion was submitted for histopathologic examination. Microscopically, the cyst wall was composed of fibrous connective tissue with extensive hemorrhage and multifocal necrosis. No epithelial or endothelial lining was identified along the luminal surface (Figure 4). Histopathologic examination revealed no cellular atypia, undifferentiated mesenchymal cell proliferation, or atypical lymphoid proliferation. Overall, these findings supported a diagnosis of hemorrhagic hepatic pseudocyst, and no histopathologic features suggestive of neoplasia were identified in the examined sections.

On postoperative day 1, the cat showed good activity and normal appetite, with no remarkable abnormalities in the monitored vital parameters. The HCT was 33.6%, with increased AST (63 U/L) and CPK (1232 U/L) activities. On postoperative day 2, the HCT was 31.8%, while AST and CPK activities decreased to 34 U/L and 397 U/L, respectively. The cat was discharged on the same day.

Following discharge, the owner reported mild lethargy for approximately 3–5 days, without vomiting, marked anorexia, or other apparent clinical abnormalities. At the 14-day postoperative recheck, the cat was clinically well, with no significant abnormalities on physical examination, CBC, or serum biochemical analysis. Notably, the HCT had increased to 48.5%. At 26 months after surgery, the owner reported that the cat had remained clinically well, and abdominal radiographs showed no evidence of a recurrent abdominal mass (Figure 5).

## 3. Discussion

The present case was diagnosed as a hemorrhagic hepatic pseudocyst based on histopathologic findings of a fibrous cyst wall with extensive hemorrhage and multifocal necrosis and the absence of an epithelial lining. Hepatic cysts have an epithelial lining, whereas pseudocysts lack an epithelial lining, consist primarily of fibrous tissue, and are generally considered acquired lesions [3,5]. Histopathologic evaluation of the cyst wall was therefore important not only for distinguishing a pseudocyst from a true hepatic cyst but also for assessing the lesion for features suggestive of neoplasia. In the present case, no cellular atypia, undifferentiated mesenchymal cell proliferation, or atypical lymphoid proliferation was identified. Overall, no histopathologic features suggestive of neoplasia were identified in the examined sections.

Previously reported hepatic cystic lesions in cats have included congenital hepatic cysts, polycystic liver disease, and vascular cysts [1,4,6,7,8,12]. Hemorrhagic hepatic cystic lesions appear to be uncommon in domestic cats, whereas hemorrhagic hepatic cysts have been described in a serval [2]. In humans, intracystic hemorrhage is also considered a rare complication of hepatic cysts [9,10,11,13]. Although its exact mechanism remains uncertain, rupture of vessels within the cyst wall associated with cyst enlargement has been proposed [10,11].

In the present case, traumatic, inflammatory, vascular, and neoplastic processes were considered as possible causes of pseudocyst formation and intracystic hemorrhage. There was no known history of trauma; however, unwitnessed trauma could not be excluded. An infectious or inflammatory contribution could not be completely excluded because cytologic examination and bacterial culture of the cystic fluid were not performed. A coagulation profile was also not performed because there were no clinical or initial hematologic findings suggestive of a systemic bleeding disorder; therefore, a potential systemic contribution to the intracystic hemorrhage could not be completely assessed. Thus, the underlying cause of pseudocyst formation and intracystic hemorrhage in this cat remained uncertain.

Another notable finding in this case was the markedly increased total bilirubin concentration in the cystic fluid (3.38 mg/dL) compared with the serum concentration (0.03 mg/dL). No gross communication between the cyst and the biliary tract was identified during surgery, making direct gross biliary leakage unlikely, although microscopic communication could not be excluded. Given the extensive intracystic hemorrhage, bilirubin derived from the degradation of erythrocyte hemoglobin within the cyst may have contributed to the increased cyst-fluid bilirubin concentration.

Hepatic cysts are often incidental findings with minimal clinical signs [1,2,6,7]. In the present case, the lesion occupied a large portion of the abdominal cavity and caused marked abdominal distension, but the cat showed only intermittent vomiting. Despite substantial intracystic hemorrhage, routine hematologic and biochemical findings at initial presentation revealed no clinically significant abnormalities. These findings suggest that a large hemorrhagic hepatic cystic lesion may be present without significant clinicopathologic abnormalities.

Radiography demonstrated the presence and extent of the abdominal cystic lesion, while ultrasonography characterized its fluid-filled appearance. CT provided more detailed information regarding the hepatic origin of the lesion and its anatomic relationship with surrounding abdominal structures. The hyperechoic region observed on ultrasonography may have represented hemorrhagic or necrotic debris, consistent with the surgical and histopathologic findings. However, imaging alone may not reliably distinguish hemorrhagic cystic lesions from other hepatic cystic lesions or cystic neoplasms [6,7,9,10,11]. Histopathologic examination was therefore important for establishing the diagnosis in this case.

Several treatment options have been described for hepatic cysts, including percutaneous aspiration, fenestration, sclerotherapy, omentalization, and surgical excision [1,2,7,8,14,15]. In human patients with hemorrhagic hepatic cysts, treatment can be selected according to the severity of clinical signs and the possibility of malignancy [11]. Conservative management with serial imaging may be considered for mildly symptomatic or asymptomatic lesions, whereas surgical intervention may be indicated for severely symptomatic lesions or when malignancy cannot be excluded [11]. Recurrence following drainage or other non-definitive treatment should also be considered when selecting an appropriate treatment approach [2,7].

In the present case, the large size of the lesion, hemorrhagic contents, and inability to establish a definitive diagnosis before surgery supported complete surgical excision. This approach allowed removal of the lesion while preserving the adjacent hepatic parenchyma and provided sufficient tissue for histopathologic examination. The cat recovered without major postoperative complications. Transient lethargy was reported after discharge. Because no additional examination was performed specifically during this period, the exact cause of the lethargy could not be determined. Postoperative discomfort or medication-related effects may have contributed to this transient finding. At the 14-day postoperative recheck, the cat was clinically well, and no clinically significant abnormalities were identified on CBC and serum biochemical analysis.

The 26-month follow-up supported a favorable long-term clinical outcome after complete surgical excision in this cat. The absence of an apparent recurrent abdominal mass on follow-up radiographs, together with the lack of owner-reported clinical concerns, provided additional information regarding the postoperative course. Nevertheless, these findings should be interpreted cautiously because follow-up assessment was limited to owner-reported clinical status and abdominal radiography rather than ultrasonography or CT. Therefore, subtle recurrence not detectable on radiographs could not be definitively excluded. Furthermore, the outcome of a single case cannot establish the long-term efficacy of surgical excision or the risk of recurrence, and additional cases with extended follow-up would be needed to better characterize long-term outcomes after surgical treatment of hepatic pseudocysts in cats.

## 4. Conclusions

This case demonstrates that hemorrhagic hepatic pseudocyst, although rare, should be included in the differential diagnosis of large hepatic cystic lesions in cats. As imaging findings alone may be insufficient to distinguish hepatic pseudocysts from other cystic lesions, the diagnosis in this case was established based on histopathologic examination. Complete surgical excision allowed removal of the large space-occupying lesion while preserving the adjacent hepatic parenchyma and provided tissue for definitive histopathologic diagnosis. The favorable postoperative course and the absence of radiographic evidence of a recurrent abdominal mass at 26 months suggest that complete surgical excision may be a treatment option for resectable hepatic pseudocysts in cats.

## Figures and Tables

**Figure 1:**
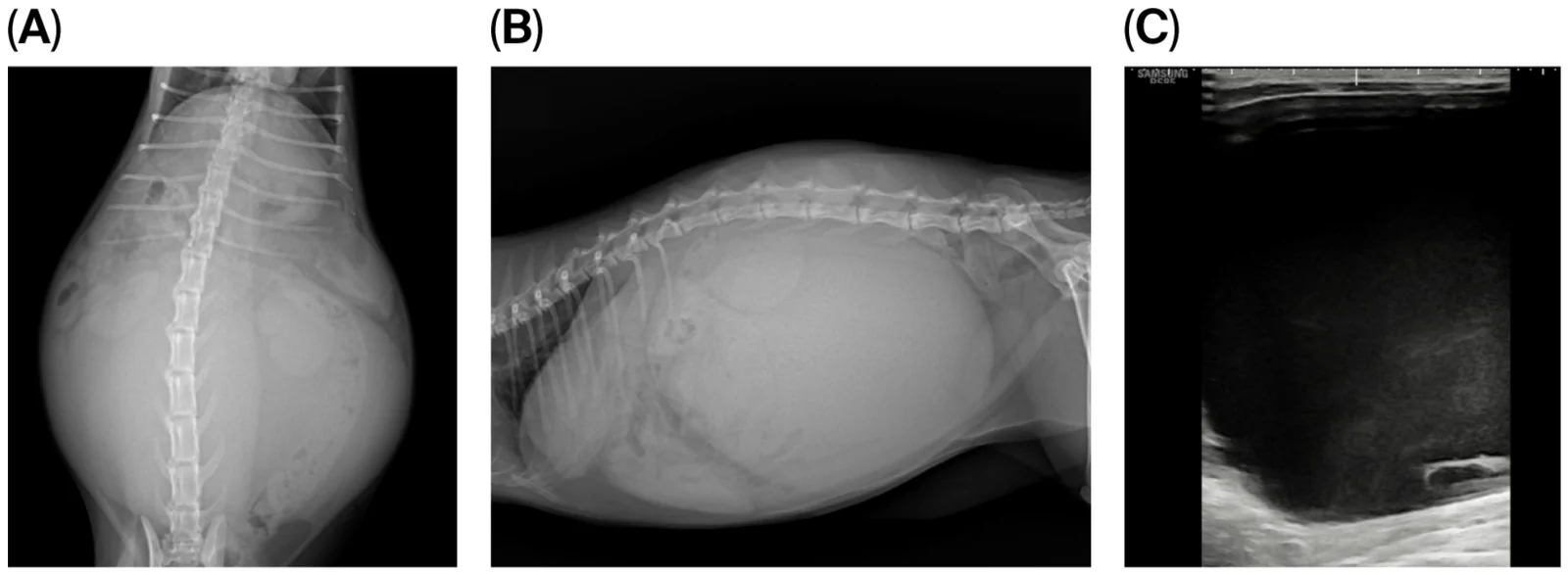
Abdominal radiographic and ultrasonographic findings. (**A**) Ventrodorsal and (**B**) right lateral abdominal radiographs showing a large, well-defined soft tissue opacity occupying most of the abdominal cavity and resulting in displacement of the gastrointestinal tract. (**C**) Abdominal ultrasonographic image showing a large fluid-filled cyst with a focal hyperechoic area within the lesion.

**Figure 2:**
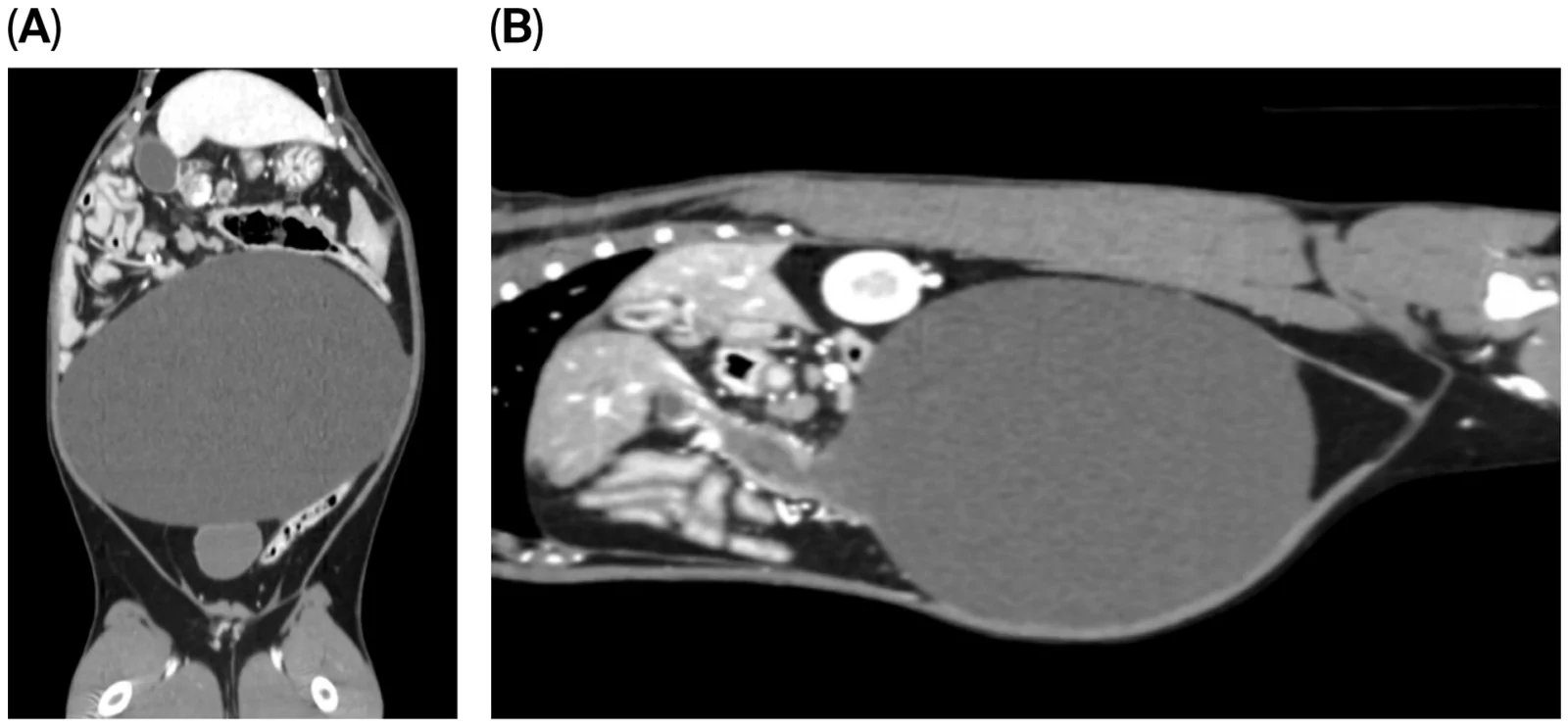
Preoperative computed tomography images in the (**A**) dorsal and (**B**) sagittal planes showing a large, well-defined cystic lesion occupying most of the abdominal cavity and causing marked displacement of the adjacent abdominal organs. The lesion showed homogeneous fluid attenuation, consistent with a hepatic cystic lesion.

**Figure 3:**
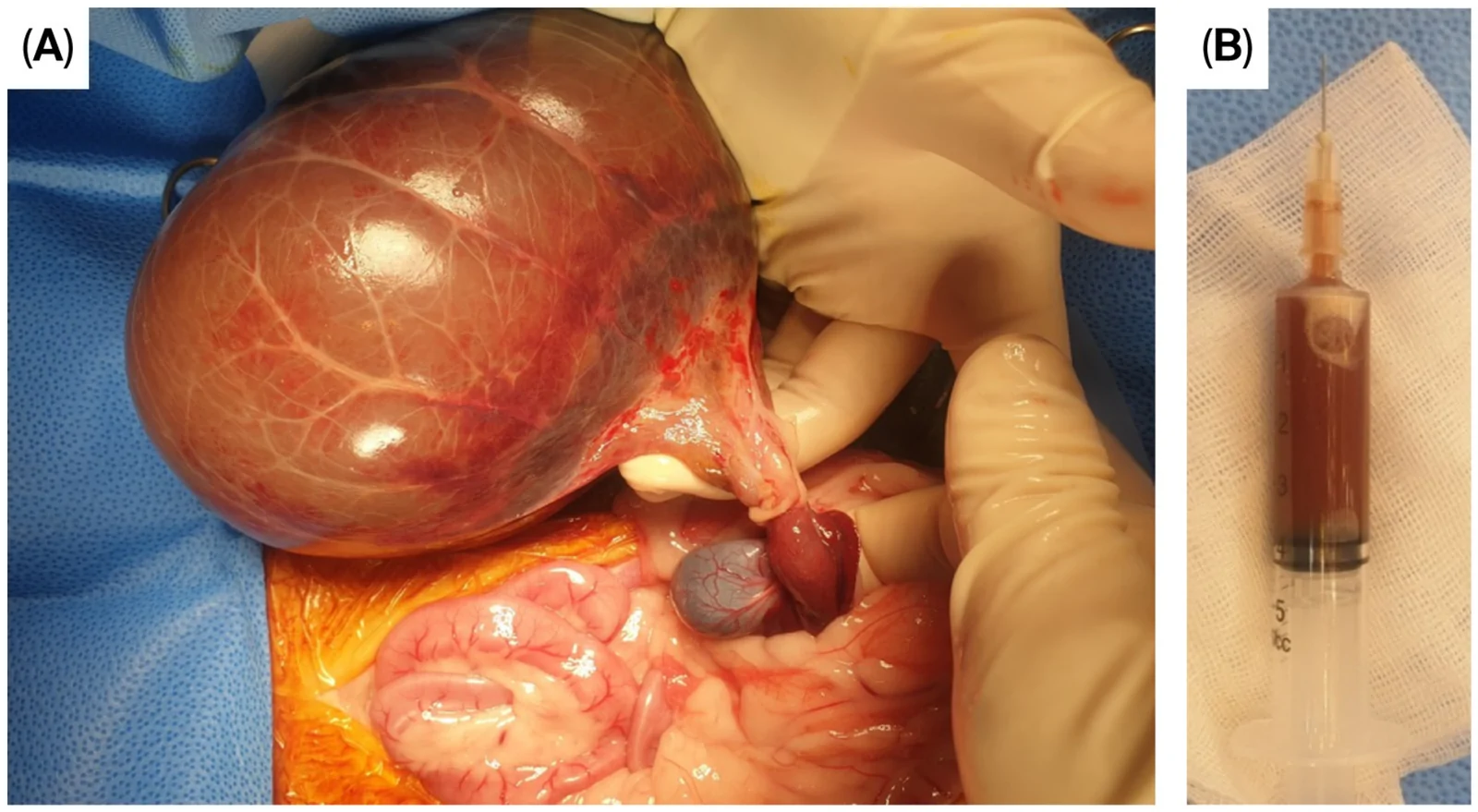
Intraoperative findings of the hepatic cyst. (**A**) A large, solitary cyst arising from the liver identified during surgical exploration. (**B**) Dark red hemorrhagic fluid aspirated from the cyst during surgery.

**Figure 4:**
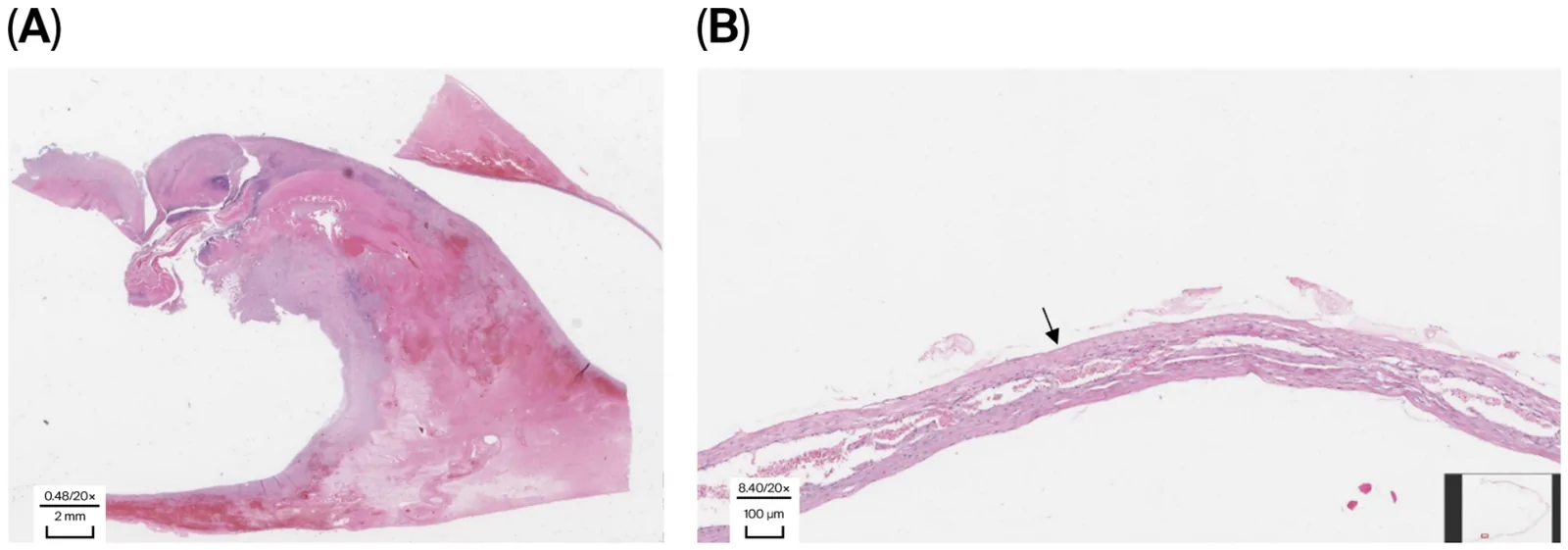
Histologic features of the hepatic pseudocyst. (**A**) Low-magnification view showing a cyst wall composed of fibrous connective tissue with extensive hemorrhage and multifocal necrosis. (**B**) Higher-magnification view of the cyst wall. The arrow indicates the luminal surface, where no epithelial lining is identified. (H&E stain, (**A**) ×40; (**B**) ×200).

**Figure 5:**
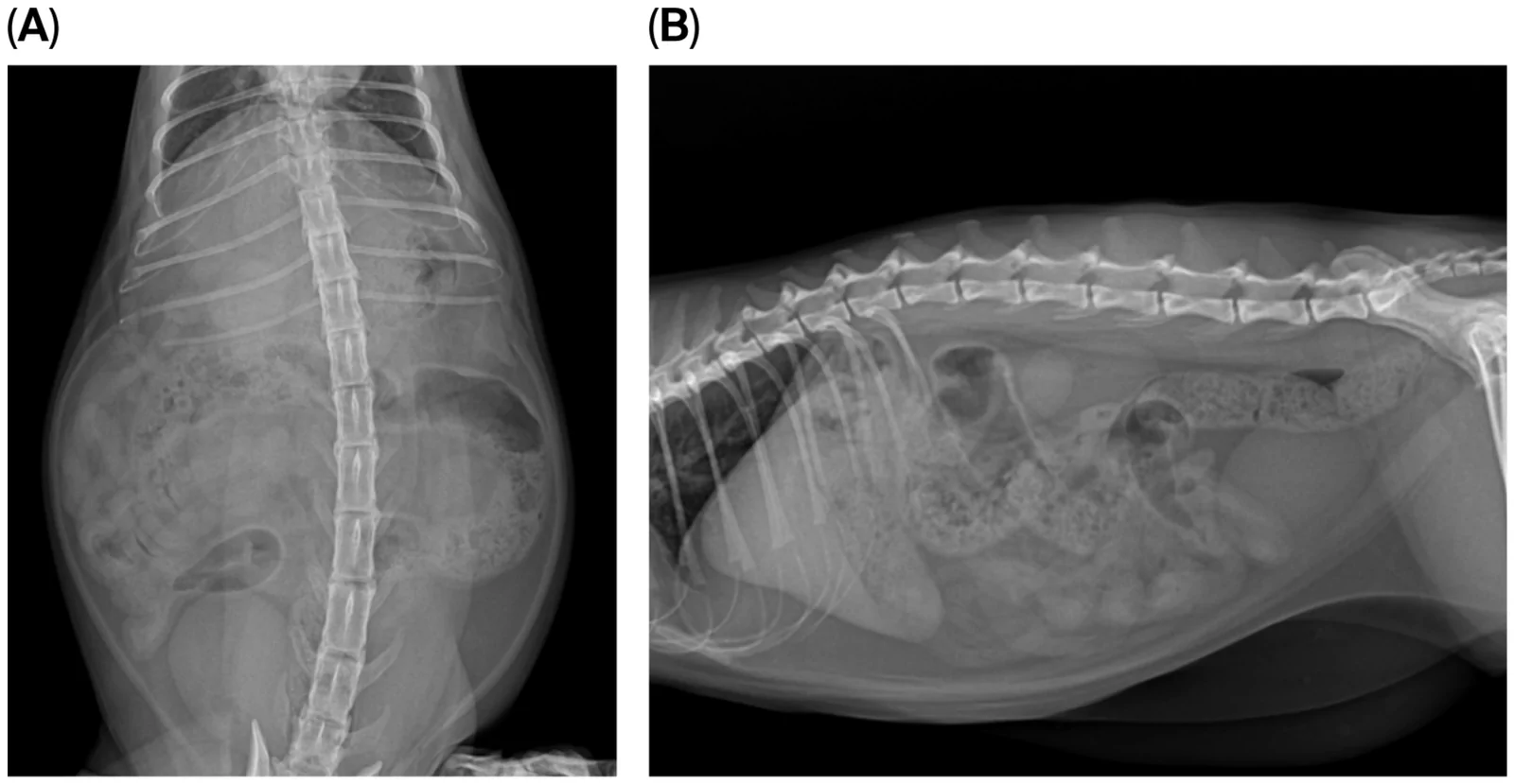
Long-term follow-up abdominal radiographs obtained 26 months after surgical excision of the hepatic pseudocyst. (**A**) Ventrodorsal and (**B**) right lateral views. No radiographic evidence of a recurrent abdominal mass was identified at the time of follow-up.

**Table 1:** Hematologic and serum biochemical findings at initial presentation.

Parameter	Result	Reference Interval
RBC (M/μL)	10.83	6.54–12.20
HCT (%)	48.3	30.3–52.3
HGB (g/dL)	15.5	9.8–16.2
RETIC (K/μL)	18.4	3.0–50.0
WBC (K/μL)	11.21	2.87–17.02
PLT (K/μL)	273	151–600
ALT (U/L)	44	12–130
AST (U/L)	34	0–48
ALP (U/L)	68	14–111
GGT (U/L)	0	0–4
TBIL (mg/dL)	0.03	0.00–0.9
ALB (g/dL)	3.5	2.2–4.0
TP (g/dL)	6.2	5.7–8.9
GLU (mg/dL)	95	74–159
BUN (mg/dL)	24.7	16.0–36.0
CREA (mg/dL)	1.35	0.80–2.40

Note: Complete blood count was performed using a ProCyte Dx hematology analyzer (IDEXX Laboratories, Inc., Westbrook, ME, USA), with the manufacturer-provided feline reference intervals. Serum biochemical analysis was performed using a DOTTO 2000 chemistry analyzer (MTD Diagnostics Co., San Nicola la Strada, Italy), with reference intervals based on the Catalyst feline adult reference intervals (IDEXX Laboratories, Inc.).

## Data Availability

The original contributions presented in this study are included in the article. Further inquiries can be directed to the corresponding author.

## Abbreviations

The following abbreviations are used in this manuscript:

RBC	red blood cell count
HCT	hematocrit
HGB	hemoglobin
RETIC	reticulocyte count
WBC	white blood cell count
PLT	platelet count
ALT	alanine aminotransferase
AST	aspartate aminotransferase
ALP	alkaline phosphatase
GGT	gamma-glutamyl transferase
TBIL	total bilirubin
ALB	albumin
TP	total protein
GLU	glucose
BUN	blood urea nitrogen
CREA	creatinine
